# Effects of the Conceptual Model of Health Literacy as a Risk: A Randomised Controlled Trial in a Clinical Dental Context

**DOI:** 10.3390/ijerph15081630

**Published:** 2018-08-01

**Authors:** Linda Stein, Maud Bergdahl, Kjell Sverre Pettersen, Jan Bergdahl

**Affiliations:** 1 Department of Clinical Dentistry, Faculty of Health Sciences, UiT—The Arctic University of Norway, 9019 Tromsø, Norway; maud@jmbergdahl.se (M.B.); janl@jmbergdahl.se (J.B.); 2 Department of Nursing and Health Promotion, Faculty of Health Sciences, OsloMet—Oslo Metropolitan University, 0130 Oslo, Norway; spetters@oslomet.no; 3 Department of Psychology, Umeå University, 901 87 Umeå, Sweden

**Keywords:** health literacy, patient education, dentistry, oral health, RCT

## Abstract

Numerous conceptual models of health literacy have been proposed in the literature, but very few have been empirically validated in clinical contexts. The aim of this study was to test the effects of the conceptual model of health literacy as a risk in a clinical dental context. A convenience sample of 133 Norwegian-speaking adults was recruited. Participants were randomly allocated to an intervention group (*n* = 64, 54% women, mean age = 50 years) and a control group (*n* = 69, 49% women, mean age = 46 years). Clinical measurements were conducted pre-intervention and six months post-intervention. In the intervention group, communication regarding patients’ oral health was tailored to their health literacy levels using recommended communication techniques, whereas the control group received brief information not tailored to health literacy levels. The ANCOVA showed significant between-group effects, finding reduced post-intervention mean gingival (*p* < 0.000) and mean plaque (*p* < 0.000) indices in the intervention group when controlling for baseline index scores. The adjusted Cohen’s d indicated large effect sizes between the intervention group and the control group for both the mean gingival index (−0.98) and the mean plaque index (−1.33). In conclusion, the conceptual model of health literacy as a risk had a large effect on important clinical outcomes, such as gingival status and oral hygiene. The model may be regarded as a suitable supplement to patient education in populations.

## 1. Introduction

Oral diseases remain a burden, despite great improvements in oral health in recent decades. Dental caries, or tooth decay due to acid-producing oral bacteria, is one of the most common preventable chronic diseases worldwide, and people are susceptible to it beginning in childhood and continuing throughout their lifetime [1]. Dental caries affects 60 to 90% of school-aged children and the vast majority of adults and accounts for oral pain, tooth loss and large expenses for both individuals and society [2]. Gingivitis, or inflammation of the gums, affects 50 to 90% of adults worldwide, depending on the precise definition considered [3]. Left untreated, gingivitis may progress to periodontitis, an inflammation below the gums and along the roots of the teeth, causing destruction of the teeth’s supporting ligaments and bone [4]. This process ultimately leads to a loosening of the teeth and potential tooth loss. Both dental caries and gingivitis are initially preventable and reversible diseases and can be halted at any stage if the bacterial biofilm covering the tooth surface, the dental plaque, is removed [3,4]. Adequate daily oral hygiene routines are important to prevent these common diseases and improve clinical status. However, this requires rather specialised skills, as well as an understanding of oral health information. In short, it requires health literacy.

Literacy and its association with health have recently gained increased attention. The WHO considers literacy to be one of the strongest predictors of health, along with age, income, employment status, education level and race or ethnic group [5]. The prevalence of limited health literacy is high, even in economically developed countries. In Europe, the European Health Literacy Project (HLS-EU) recently reported limited health literacy among 47% of respondents in its international survey [6]. Systematic reviews found that limited health literacy is associated with poor health outcomes across different diseases, greater difficulty participating in shared decision-making, less understanding regarding the importance of preventive behaviour and poorer self-management of disease [7,8].

Oral health literacy is defined as the degree to which individuals have the capacity to obtain, process and understand basic oral health information and services needed to make appropriate health decisions [9]. Importantly, oral health literacy has been identified as a potential barrier to effective disease prevention, diagnosis and treatment [10]. The burden of limited health literacy in different health contexts is considered enormous, and the potential to reduce poor outcomes through intervention has been emphasised [11]. The literature has presented numerous conceptual models of health literacy, though very few have been empirically validated [12]. The central tenet is that identifying low levels of health literacy will foster the implementation of tailored interventions to improve health outcomes [7,13]. In a paper on the evolving concept of health literacy, Nutbeam presented two conceptual models inspired by several previously developed models [13]. One of the models positions health literacy as a risk factor that needs to be identified and appropriately managed in clinical care, while the other positions health literacy as an asset to be built and an outcome to health education. To the best of our knowledge, no clinical trial has yet been published assessing the effects of the risk model on clinical outcomes, such as gingival status or oral hygiene. Thus, the objective of this study was to test the effects of the conceptual model of health literacy as a risk in a clinical dental setting. Based on the model, we hypothesised that patients provided with communication sensitive to oral health literacy would improve their gingival status and oral hygiene.

## 2. Materials and Methods

### 2.1. Study Design

The study was designed as a randomised, examiner- and participant-blinded, controlled clinical trial. Clinical oral health measurements to assess oral hygiene and gingival health were conducted pre-intervention and six months post-intervention. To be eligible for inclusion, participants had to be older than 20 years, have no severe visual impairments, and have mastery of the Norwegian language. Participants were recruited from a list of adults who had volunteered to be enrolled as dental students’ patients at the University Dental Clinic, but had not yet been diagnosed or started any form of dental treatment. Due to the large age differences among eligible individuals, a stratified randomisation was considered necessary to balance the control and intervention groups with respect to age. Pair matching across groups was performed using gender and an age range of five years. The allocation was concealed by having a person who had no information regarding the participants’ oral health and was not a member of the research team perform the randomisation procedure. To detect medium-size effects (Cohen’s d = 0.5) with a power of 0.80 (α = 0.05, two-tailed), an a priori sample size power calculation was conducted using the software G*Power 3 [14] (Institut für Experimentelle Psychologie, Dusseldorf, Germany). The calculation indicated that a sample size of 64 participants per group was required. We followed the CONSORT guidelines to properly design, conduct and report the clinical trial. The study was registered in the international online database clinicaltrials.gov (ID: NCT 01118143). Ethical approval was granted by the Regional Ethical Committee for Medical and Health Research, Tromsø, Norway (2010/31-11), and the study was conducted in accordance with the World Medical Association Declaration of Helsinki.

### 2.2. Trial Procedure

Individuals who returned signed consent forms by mail after receiving written information about and invitations to participate in the study were invited to the dental clinic at the Public Dental Service Competence Centre of Northern Norway, Tromsø, Norway, for study participation. As inadequate comprehension of informed consent is common among study participants [15], the study information was repeated orally, and efforts were made to make sure the participants understood both the advantages and disadvantages of the study, that participation was voluntary and that their decision would not affect their future care at the University Dental Clinic [16]. Participants were allocated to either the intervention or the control group prior to the pre-intervention appointment. All pre-intervention characteristics were collected during the same visit. First, oral health literacy was assessed. Second, clinical examinations were performed. Interventions took place immediately after the clinical measurements. Finally, participants filled out a self-administered questionnaire. Post-intervention measurements were scheduled six months after the pre-intervention measurements. The recruitment of participants started in May 2010 and ended in June 2011, when the number of participants reached that required by the power calculation. The data collection period lasted from June 2010, when the first baseline measurements were conducted, until the last follow-up measurements were completed in February 2012.

### 2.3. Measurements

Oral health literacy (range: 1 to 5) was assessed using the Adult Health Literacy Instrument for Dentistry (AHLID) [17]. The AHLID is a newly developed and validated Norwegian interview instrument. It consists of printed oral health information texts, medicine prescriptions, post-treatment information and brochures on dental diseases, all frequently used for the benefit of adult dental patients to complement communication with dental professionals. Participants were asked to read ten different health information texts and answer questions related to their content. The texts and accompanying questions corresponded to five different levels of oral health literacy ranging from 1 (lowest) to 5 (highest). The AHLID interview guide was used to score the participants according to these levels. The interviews took place in a suitable room free from disturbing noises and dental equipment. All AHLID interviews were conducted by the same dental researcher, who was also the principal investigator.

Clinical parameters included dentition status, gingival status and oral hygiene status. Dental status was examined using the WHO criteria to account for the numbers of decayed, missing and filled teeth (DMFT) [18]. The gingival index [19] was utilised to objectively measure gingival status, and the plaque index [20] was used to objectively measure oral hygiene status. These indices are well known and have been used worldwide since the 1960s. Mean gingival and mean plaque index scores for each participant were obtained by registering four tooth surfaces (distal, buccal, mesial and lingual/palatal) on all present teeth, except third molars. For oral hygiene, the amount of plaque was measured, and for gingival status, the amount of bleeding for these sites was measured. Importantly, for both indices, scores from the four sites of all teeth were added and then divided by the present number of teeth to create the mean variables for oral hygiene and gingival bleeding, according to the method described by Silness and Löe [19,20]. Background variables, such as gender, age, income, education level and smoking status, were collected using a self-administered questionnaire. The same trained dental hygienist performed all clinical examinations on all participants pre- and post-intervention. The dental hygienist was calibrated by a dentist and senior clinical researcher to minimise measurement errors. To examine reproducibility of the indices, plaque and gingival indices were calculated by registering scores obtained by the dental hygienist who performed all analyses and the dentist/senior researcher prior to the study. For this intra-examiner reliability measure, six index teeth in 10 volunteering individuals, not involved in the study, were assessed. The kappa coefficient scores were 0.70 for gingival index and 0.78 for plaque index, representing substantial agreement. The dental hygienist was blinded to group allocation and participants’ oral health literacy levels. The primary study outcome variable was the mean gingival index score, and its secondary outcome variable was the mean plaque index score.

### 2.4. Interventions

#### 2.4.1. Communication According to the Model (Intervention Group)

**Figure 1:**
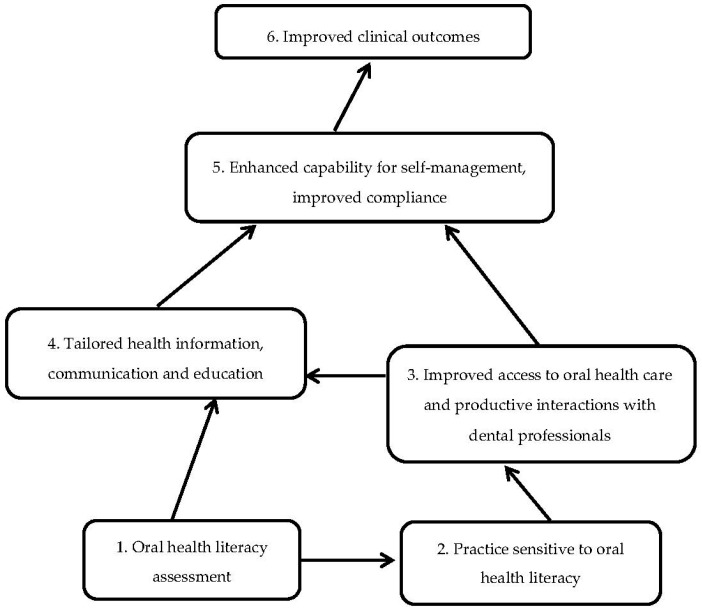
Model for the study adapted to oral health literacy from the Conceptual model of health literacy as a risk, proposed by Nutbeam [13].

For participants in the intervention group, communication regarding their gingival status and oral hygiene was carried out according to the conceptual model of health literacy as a risk [14] (Figure 1). Utilising this model, the first step was an assessment of health literacy. A clinical environment sensitive to health literacy was emphasised by considering the participants’ health literacy level, as the clinician’s sensitivity can improve patients’ access to health care and enhance the quality of patient–clinician interactions. As a consequence, the clinician was more skilled to provide patient education that was tailored to individual needs and capacities. Tailored communication increases patients’ capability for self-management, which in turn may lead to improved clinical outcomes. Health literacy tools are typically organised into four categories: Improving spoken communication, improving written communication, improving self-management and empowerment and improving supportive systems [21]. Communication techniques utilised in the intervention group included speaking in plain, non-medical language; confirming understanding using the “teach-back’’ approach by having patients repeat information back in their own words; and showing patients how to operate dental devices. Furthermore, open-ended questions were used to avoid yes/no answers [22,23]. Radiographs, pictures and models of teeth and jaws were used as visual supplements to the oral conversations when considered necessary for comprehension [24]. Because the effect of printed or written health information materials is considered greater when the information is personalised [25], all participants in the intervention group were provided with an individualised take-home message. Inspired by the “Ask me three” approach [26], the take-home message concentrated on three important questions: “What is my main problem?”, “What do I need to do?” and “Why is it important for me to do this?” The written message was written in bullet points. The participants were provided with the recommended oral hygiene devises free of charge. The same person who conducted all AHLID interviews also performed the interventions, which lasted from 3 to 10 min each.

#### 2.4.2. General Communication (Control Group)

Participants in the control group received information regarding their gingival status and oral hygiene according to standard practice in general dentistry. Brief information was delivered orally, and no written information was provided. The communication was not sensitive to oral health literacy and did not follow the conceptual model of health literacy as a risk. The same person who conducted all AHLID interviews and performed the interventions for the intervention group also communicated with the control group. The oral communications lasted for about 3 to 4 min each.

### 2.5. Statistical Analyses

Descriptive statistics were performed on the pre-intervention characteristics of the study participants in both groups. Differences between the intervention and control groups were tested with an independent sample *t*-test for continuous variables and a chi-square test for categorical variables. Paired sample *t*-tests were performed on both groups separately to investigate within-group effects on the pre-intervention and post-intervention mean gingival index and mean plaque index. An analysis of covariance (ANCOVA) was used to evaluate between-group treatment effects on mean gingival and plaque index. Pre-intervention measures were entered as covariates to adjust for differences between groups in pre-intervention scores. Within- and between-group effect sizes were measured using Cohen’s d. A Cohen’s d of 0.2 was considered a small effect, 0.5 was considered a medium effect and 0.8 was considered a large effect [27]. *p*-Values lower than 0.05 were considered statistically significant. Statistical analyses were performed using IBM SPSS Statistics software for Windows (version 21.0, IBM SPSS Inc., Chicago, IL, USA). Within-group effect sizes were calculated using Becker’s effect size calculator separately for the mean gingival index and the mean plaque index [28]. Between-group effect sizes were calculated separately for the mean gingival index and the mean plaque index using the adjusted mean difference between the intervention group and the control group divided by the estimated pooled standard deviation obtained from the square root of the MS error of the ANCOVA model. 

## 3. Results

**Figure 2:**
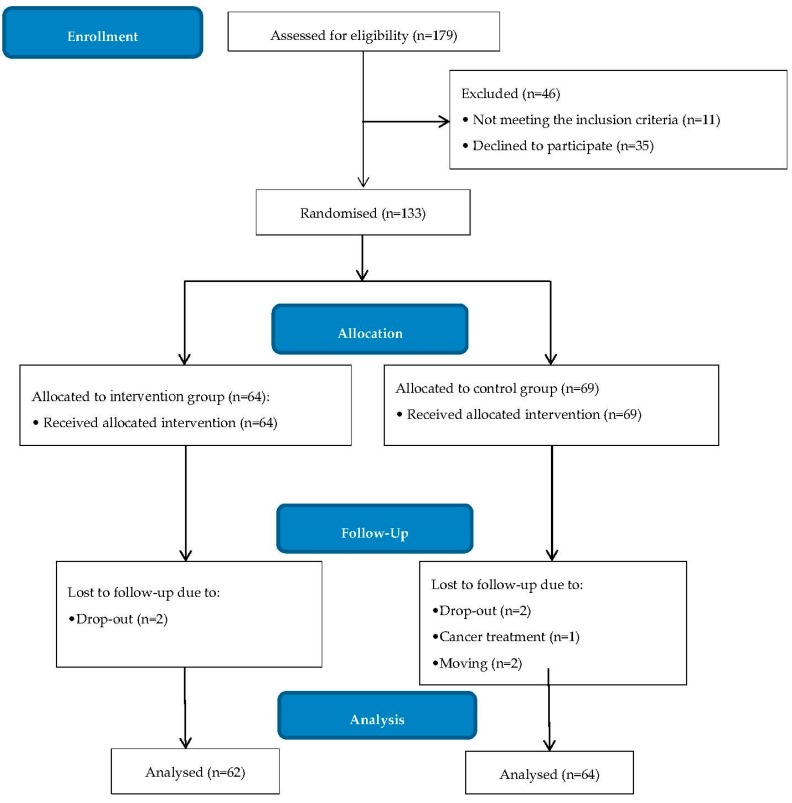
Flow chart of study participants.

A total of 133 adults were randomly allocated for study participation: 64 in the intervention group and 69 in the control group. Two participants from each group were lost to follow-up due to drop-outs, and an additional three participants were lost in the control group due to other reasons (Figure 2). All participants were analysed in the group to which they were randomised. The participants’ pre-intervention characteristics are presented by group allocation in Table 1. There were no significant baseline differences between the groups with respect to gender, age, level of education, oral health literacy level, smoking status, chronic disease, DMFT or plaque index. However, the intervention group had a significantly higher mean gingival index (*p* < 0.001). Paired-sample *t*-tests showed that the mean gingival index decreased significantly from the pre-intervention to the post-intervention measurement in the intervention group (*p* < 0.000), but not in the control group (*p* = 0.480). Regarding the mean plaque index, a significant decrease was seen in both the intervention group (*p* < 0.000) and the control group (*p* < 0.000) (Table 2). The within-group effect size for the gingival index was zero in the control group, but large in the intervention group. The plaque index effect size was large in the intervention group and small in the control group (Table 2). The ANCOVA showed a significant between-group effect, indicating that reduction in both the post-intervention mean gingival index and the mean plaque index was significantly greater for the intervention group than the control group when controlling for baseline index scores (Table 3). The adjusted Cohen’s d indicated large between-group effect sizes for both the mean gingival index and the mean plaque index.

## 4. Discussion

The hypothesis of this study was that patients provided with communication sensitive to oral health literacy according to the conceptual model of health literacy as a risk would improve their gingival status and oral hygiene compared to the control group. Our findings support the hypothesis. The participants’ pre-intervention characteristics (Table 1) reveal that the intervention group had a higher mean gingival index score. This can be seen as a weakness of the study; however, an ANCOVA analysis was used to adjust these baseline differences. A significant post-intervention reduction in the gingival index score was observed in the intervention group, but not in the control group. Both groups exhibited a significant decrease in the mean plaque index, although the decrease was more pronounced in the intervention group. We interpret the decrease in the plaque index for the control group as a non-specific effect of trial participation due to the consequence of being observed (i.e., the Hawthorne effect). Although the Hawthorne effect should not affect the assessment of the difference between intervention and control, it may result in an inflated estimate of effect size in routine clinical settings due to an over-estimation of the responses from both groups [29]. All participants were aware that participation involved a repeated measurement of oral health variables. It might be reasonable to assume that some participants payed extra attention to and spent more time on their oral hygiene on the day of measurement, resulting in less plaque. Gingival status is a more reliable measure of sustained behaviour over time, since the presence of bacterial plaque in contact with the gingiva will cause gingivitis if not removed on a regular basis. The post-intervention measure showed a significant reduction in the gingival index score in the intervention group, but not the control group. This result indicates that communication sensitive to oral health literacy can effectively improve oral health outcomes, as hypothesised. The improved oral health was likely due to enhanced capability for self-management and motivation, leading to improved compliance [13].

A recent review of oral health promotion trials in relation to oral hygiene and gingival health found no clear indication that any particular type or style of educational approach was more effective than others [30]. However, no oral health literacy interventions had been published at the time of this study; therefore, they are not included in the review. Since the study, a handful of oral health literacy intervention studies have been published. Ju et al. [31] tested the efficacy of an oral health literacy intervention based on Bandura’s Social Cognitive Theory to enhance oral health literacy among indigenous Australian adults and concluded that the intervention was partially successful in improving oral health literacy and oral health-literacy related outcomes after multiple imputations. Vilella et al. [32] evaluated the effect of oral health literacy on the retention of health information in pregnant women. The results suggested that low oral health literacy has a negative effect on information retention, but that only spoken oral health interventions can address differences in literacy levels. These studies provide new knowledge of vulnerable populations, but neither includes clinical outcomes. In the broader health context, a systematic review of interventions designed to mitigate the effect of low health literacy found that common features of interventions that changed distal outcome (i.e., biomarkers of disease) had a solid theoretical basis, emphasised skill-building and were delivered by a health professional [33]. Our study included these features, and the results regarding distal outcomes (i.e., the gingival index and the plaque index) support the design of health literacy studies. Furthermore, most of the interventions included in the systematic review occurred in a single session focused on making health information more understandable to patients with low health literacy, which used visual aids and/or handouts with materials written in simpler language to complement conversations between health professionals and their patients [30]. Participants in the intervention group were provided with these communication techniques during the dental encounter. All techniques were well known and should be easy to conduct in a clinical dental setting. However, a recent national survey from the U.S. concluded that the routine use of communication techniques, including some techniques thought to be most effective for patients with limited literacy skills, is low among dentists [34]. Another study assessing dental hygienists’ communication techniques found routine use of only one-third of the recommended communication techniques for patients with low health literacy [35]. These studies support the need to train health professionals in communicating with patients with low health literacy. Furthermore, it has been argued that the barriers caused by limited health literacy in a clinical context may be as much a problem of insufficient clinician competence to reduce unnecessary complexity and improve communication skills as they are a problem of limited health literacy skills in patients [36]. Implementing clinical practice sensitive to patients’ health literacy, as proposed in the conceptual model of health literacy as a risk, might be an important step to reduce these barriers in clinical encounters.

The strengths of this study include its Randomised controlled trial (RCT) design and good follow-up rates. Limitations include its convenience sample of persons seeking care at a university dental clinic. Compared to the general population, these individuals may be more interested in oral health and more motivated to participate in a study, which may influence the results. At baseline, the intervention group had a higher mean gingival index than the control group, which may be seen as a weakness in the randomisation process. The groups were randomised using age and gender stratifications. Since periodontal problems typically increase with age, the age stratification was used, but there were still differences in the gingival index. In future studies, periodontal status might be considered in the stratification. Longer follow-up times and additional repeated measurements would also have been beneficial; however, such measures were limited due to a lack of resources. The drop-out rate was similar and low for both groups, which can be interpreted as indicating that the study instructions were followed and that both groups received the same care. The participants were blinded to group allocation, but the researcher who performed the interventions was not. This fact could have influenced the results in favour of the intervention group. Most importantly, however, the clinical examiner was blinded to group allocation, which is a strength of the study. Another strength is that both groups received the intervention from the same researcher, resulting in more consistent interpersonal interactions. This may have reduced the unwanted effects of the intervention. On the other hand, one cannot be sure of whether the positive effects achieved in this study on gingival status and oral hygiene in the intervention group are due exclusively to the approaches made. For instance, a study from the dental context [37] demonstrated associations among patients’ degree of self-efficacy, their oral health literacy and their dental neglect (negative correlation). Nutbeam [13], thus, commented in his conceptual model that actions to improve health literacy should be focused on developing age- and context-specific health knowledge and that strong self-efficacy is necessary to implement increased health knowledge in ways that enable people to exert greater control over their health and health-related decisions. Therefore, the patients’ degree of self-efficacy should have favourably been assessed in this study. On the other hand, it would be reasonable to include health literacy in intervention studies testing other models. One editorial emphasised that oral health researchers must develop and test new theories and models instead of conducting interventions based on the same well-established theories [38]. Furthermore, a recent systematic review of psychological approaches to behaviour change for improved oral hygiene concluded that understanding the benefits of behaviour change and the seriousness of the disease are important predictors for the likelihood of behaviour change [39]. 

The model on which we based this intervention focuses on the direct pathways between health literacy and health outcomes. We do recognise that other circumstances may mediate the effects of the intervention. Conceptualising health literacy as an individual risk does have limitations and improving health literacy in a population involves more than the transmission of health information, although this certainly remains a fundamental task [13]. Focusing on individual behavioural change and managing barriers to health literacy in a clinical context are important; however, clinical approaches alone can never be sufficient to enhance oral health in populations. Upstream public health actions are also required.

## 5. Conclusions

To the best of our knowledge, this is the first randomised control trial to test the effects of the conceptual model of health literacy as a risk in a clinical dental context. Our findings should, therefore, be seen a first step to provide evidence, and we hope that they will encourage other researchers to perform similar studies. In conclusion, the conceptual model of health literacy as a risk demonstrates a significant improvement in important clinical outcomes, such as gingival status and oral hygiene, in adult patients. The hypothesis that patients provided with communication sensitive to oral health literacy will improve their gingival status and oral hygiene was supported. The model may be considered a suitable clinical supplement to health literacy-based patient education in populations.

## Figures and Tables

**Table 1:** Pre-intervention characteristics of study participants.

Characteristics	Intervention Group (*n* = 64)	Control Group (*n* = 69)	*p*-Value ^a^
Gender			0.394
Men	46%	51%	
Women	54%	49%	
Age (years) ^b^	49.53 ± 14.97	46.35 ± 14.23	0.211
Education level			0.917
Elementary school	19%	16%	
High school	36%	38%	
University/University college	45%	46%	
Oral health literacy level ^b^	3.02 ± 0.72	2.96 ± 0.88	0.675
Smoker	15%	27%	0.095
Chronic disease	45%	44%	0.832
DMFT ^b^	18.19 ± 6.62	17.94 ± 6.36	0.828
Gingival index ^b^	1.34 ± 0.29	1.16 ± 0.35	0.001
Plaque index ^b^	0.53 ± 0.36	0.48 ± 0.36	0.420

^a^ Independent sample *t*-test for continuous data. Chi-square test for categorical data; ^b^ Values are mean ± DS.

**Table 2:** Within-group measurements and effects of primary and secondary outcome variables.

Group	Pre-Intervention Gingival Index ^a^	Post-Intervention Gingival Index ^a^	*p*-Value ^b^	Cohen’s d	Pre-Intervention Plaque Index ^a^	Post-Intervention Plaque Index ^a^	*p*-Value ^b^	Cohen’s d
Intervention (*n* = 62)	1.34 ± 0.29	0.72 ± 0.42	0.000	−1.775	0.53 ± 0.36	0.08 ± 0.13	0.000	−1.663
Control (*n* = 64)	1.16 ± 0.35	1.12 ± 0.46	0.480	−0.098	0.48 ± 0.36	0.34 ± 0.35	0.000	−0.394

^a^ Values are mean ± SD. ^b^ *p*-values obtained from paired-sample t-tests performed for separate groups.

**Table 3:** Between-group effects of interventions on primary and secondary outcome variables.

Outcome Variable	Group	ANCOVA-Adjusted Mean (95% CI)	MS Error	*p*-Value	Adjusted Cohen’s d
Gingival index			0.190	0.000	−0.98
	Intervention (*n* = 62)	0.70 (0.591–0.815)			
	Control (*n* = 64)	1.13 (1.020–1.242)			
Plaque index			0.045	0.000	−1.33
	Intervention (*n* = 62)	0.07 (0.019–0.125)			
	Control (*n* = 64)	0.35 (0.294–0.399)			

## References

[1] Kidd E., Fejerskov O. (2016). Essentials of Dental Caries.

[2] Petersen P.E., Bourgeois D., Ogawa H., Estupinan-Day S., Ndiaye C. (2005). The global burden of oral diseases and risks to oral health. Bull. World Health Organ..

[3] Pihlstrom B.L., Michalowicz B.S., Johnson N.W. (2005). Periodontal diseases. Lancet.

[4] Selwitz R.H., Ismail A.I., Pitts N.B. (2007). Dental caries. Lancet.

[5] World Health Organization Health Literacy. The Solid Facts. http://www.thehealthwell.info/node/534072.

[6] HLS-EU Consortium Comparative Report of Health Literacy in Eight EU Member States. The European Health Literacy Project. http://www.healthliteracy.ie/wp-content/uploads/2012/09/HLS-EU_report_Final_April_2012.pdf.

[7] Berkman N.D., Sheridan M.D., Donahue M.D., Halpern D.J., Crotty K. (2011). Low health literacy and health outcomes: An updated systematic review. Ann. Int. Med..

[8] Easton P., Entwistle V.A., Williams B. (2010). Health in the hidden population of people with low literacy. A systematic review of the literature. BMC Public Health.

[9] U.S. Department of Health and Human Services (2003). A National Call to Action to Promote Oral Health.

[10] Podschun G. (2012). National plan to improve health literacy in dentistry. J. Calif. Dent. Assoc..

[11] U.S. Department of Health and Human Services National Action Plan to Improve Health Literacy. http://www.health.gov/communication/hlactionplan/.

[12] Sørensen K., Van den Broucke S., Fullam J., Doyle G., Pelikan J., Slonska Z., Brand H. (2012). Health literacy and public health: A systematic review and integration of definitions and models. BMC Public Health.

[13] Nutbeam D. (2008). The evolving concept of health literacy. Soc. Sci. Med..

[14] Faul F., Erdfelder E., Lang A.-G., Buchner A. (2007). G*Power 3: A flexible statistical power analysis program for the social, behavioral, and biomedical sciences. Behav. Res. Methods.

[15] Jackson R.D., Echert G.J. (2008). Health literacy in an adult dental research population: A pilot study. J. Public Health Dent..

[16] Sugarman J., Paasche-Orlow M. (2006). Confirming comprehension of informed consent as a protection of human subjects. J. Gen. Intern. Med..

[17] Stein L., Pettersen K.S., Bergdahl M., Bergdahl J. (2015). Development and validation of an instrument to assess health literacy in Norwegian adult dental patients. Acta Odontol. Scand..

[18] World Health Organization (2000). WHO Oral Health Country/Area Profile Programme (CAPP).

[19] Silness J., Löe H. (1964). Periodontal disease in pregnancy II. Correlation between oral hygiene and periodontal condition. Acta Odontol. Scand..

[20] Löe H., Silness J. (1963). Periodontal disease in pregnancy I. Prevalence and severity. Acta Odontol. Scand..

[21] Tapp H., Dunlin M., Plescia M., Daaleman T., Herlton M. (2018). Chronic disease self-management. Chronic Illness Care.

[22] Tamura-Lis W. (2013). Teach-back for quality education and patient safety. Urol. Nurs..

[23] Schillinger D., Piette J., Grumbach K., Wang F., Wilson C., Daher C., Leong-Grotz K., Castro C., Bindman A.B. (2003). Closing the loop. Physician communication with diabetic patients who have low health literacy. Arch. Intern. Med..

[24] Houts P.S., Doak C.C., Doak L.G., Loscalzo M.J. (2006). The role of pictures in improving health communication: A review of research on attention, comprehension, recall and adherence. Patient Educ. Couns..

[25] Haynes R.B., Ackloo E., Sahota N., McDonald H.P., Yao X. (2008). Interventions for enhancing medication adherence. Cochrane Database Syst. Rev..

[26] DeWalt D.A., Broucksou K.A., Hawk V., Brach C., Hink A., Rudd R., Callahan L. (2011). Developing and testing the health literacy universal precautions toolkit. Nurs. Outlook.

[27] Cohen J. (1988). Statistical Power Analysis for the Behavioral Sciences.

[28] Becker L.A. Becker’s Effect Size Calculator. http://www.uccs.edu/~lbecker/.

[29] McCarney R., Warner J., Iliffe S., van Haselen R., Griffin M., Fisher P. (2007). The Hawthorne effect: A randomized, controlled trial. BMC Med. Res. Methodol..

[30] Watt R.G., Marinho V.C. (2005). Does oral health promotion improve oral hygiene and gingival health?. Periodontol.

[31] Ju X., Brennan D., Parker E., Mills H., Kapellas K., Jamieson L. (2017). Efficacy of an oral health literacy intervention among indigenous australian adults. Commun. Dent. Oral Epidemiol..

[32] Vilella K.D., Fraiz F.C., Benelli E.M., Assunção L.R. (2017). Oral health literacy and retention of health information among pregnant women: A randomised controlled trial. Oral Health Prev. Dent..

[33] Sheridan S.L., Halpern D.J., Viera A.J., Berkman N.D., Donahue K.E., Crotty K. (2011). Interventions for individuals with low health literacy: A systematic review. J. Health Commun..

[34] Rozier R.G., Horowitz A.M., Podschun G. (2001). Dentist-patient communication techniques used in the United States: The results of a national survey. J. Am. Dent. Assoc..

[35] Flynn P., Schwei K., VanWormer J., Skryzpcak K., Acharya A. (2016). Assessing dental hygienists’ communication techniques for use with low oral health literacy patients. J. Dent. Hyg..

[36] Paasche-Orlow M.K., Wolf M. (2010). Promoting health literacy research to reduce health disparities. J. Health Commun..

[37] Lee J.Y., Divaris K., Baker A.D., Rozier R.G., Vann W.F. (2012). The relationship of oral health literacy and self-efficacy with oral health status and dental neglect. Am. J. Public Health.

[38] Noar S.M. (2011). Letter to the editor: Charting the course forward: Promising trends in health behavior theory application. J. Public Health Dent..

[39] Newton T.J., Asimakopoulou K. (2015). Managing oral hygiene as a risk factor for periodontal disease: A systematic review of psychological approaches to behaviour change for improved plaque control in periodontal management. J. Clin. Periodontol..

